# A detaching, V-shaped tibial tubercle osteotomy is a safe procedure with a low complication rate

**DOI:** 10.1007/s00402-020-03375-w

**Published:** 2020-02-28

**Authors:** Akkie Rood, Jordy van Sambeeck, Sander Koëter, Albert van Kampen, Sebastiaan A. W. van de Groes

**Affiliations:** 1 Department of Orthopaedics, Radboud University Medical Centre, Geert Grooteplein Zuid 10, 6500 HB Nijmegen, The Netherlands; 2 Department of Orthopaedics, Canisius Wilhelmina Hospital, Nijmegen, The Netherlands

**Keywords:** MPFL, Patellofemoral instability, Tibial tubercle, TTO

## Abstract

**Introduction:**

In patients with recurrent patellar dislocations, a tibial tubercle osteotomy (TTO) can be indicated to correct patella alta or an increased trochlear groove–tibial tubercle distance. Several surgical techniques are described. Previous studies emphasize that detaching osteotomies results in devascularisation, which can lead to non-union and tibial shaft fractures. The aim of this study was to report the complication rates directly related to the surgical technique of a V-shaped TTO, where the tubercle is completely released from its periosteum using a step-cut osteotomy.

**Methods:**

The retrospective case series comprised a large cohort of 263 knees with patella alta in 203 patients who underwent a V-shaped TTO, with or without additional realignment procedures, between March 2004 and October 2017. Data were obtained from available patient files. Complications were defined as minor or major.

**Results:**

Thirteen major complications were registered (4.9%) including two tibial fractures (0.75%) and one non-union (0.37%). Five complications (1.9%) were defined as minor. Removal of the screws because of irritation or pain was seen in 22 cases (8.2%).

**Conclusion:**

A V-shaped TTO is a safe procedure. The presumed higher risk for tibial fractures or pseudo-arthrosis could not be confirmed.

## Introduction

Patellofemoral instability is a common problem in adolescents (2–31:100,000). In case of recurrent patellar instability, surgical management results in a lower risk of recurrent dislocation than conservative management [1]. TTO is indicated in patients with recurrent patellar dislocations due to patella alta or an increased tibial tubercle–trochlear groove (TT–TG) distance. Several types of osteotomies are described: the modified Elmslie–Trillat medialisation technique [2], the Fulkerson anteromedialisation technique [3], a sliding TTO [4], and techniques in which the tibial tuberosity is completely detached [5]. A systematic review by Payne et al. concluded that the risk of complications is related to the employed technique [6]. In their review, the complication rate lies between 3.3 and 10.7%. When performing a V-shaped TTO, the tibial tubercle with periosteum is completely detached from the tibia and a step-cut osteotomy is used [7]. Some authors suggest that maintaining the medial and/or distal periosteum at the tubercle when performing an osteotomy is crucial for preserving the vascularisation and osteotomy union [8, 9]. Also, the theory of creating a tibial stress fracture when using a step-cut osteotomy lives among surgeons [10]. Payne et al. stated that osteotomies that involve complete detachment of the tubercle have an increased risk of non-union and tibial fractures compared with those in which a distal cortical hinge is maintained [6]. 

However, the hypothesized advantages of the V-shaped TTO are that the risk on non-union is low due to the triangular shape of the bone block with a twice as big bone contact area of the trabecular bone, and the intrinsically stable nature of the shape of the osteotomy in comparison to a sliding flat osteotomy. Only small sample size studies have been performed on this subject to the best of our knowledge [5, 11]. Large studies reporting the complication rates of a V-shaped TTO are missing, but necessary, to give a clearer view on this and can help to determine the optimal technique. The aim of this study was to report the complication rates directly related to the surgical technique of a V-shaped TTO, where the tubercle is completely released from its periosteum using a step-cut osteotomy.

## Materials and methods

### Data collection

All patients operated between March 2004 and October 2017 in the Radboud University Medical Centre, Nijmegen, using a V-shaped TTO were included. The indication for a tibial tubercle transfer was the recurrent patellar dislocations in combination with a patella alta (Caton–Deschamps index > 1.2), as underlying anatomical risk factor, after the failure of conservative management with or without an increased TT–TG distance. Two experienced surgeons using a similar surgical technique performed all the procedures. Additional simultaneous procedures were performed if indicated, such as medial patellofemoral ligament (MPFL) reconstruction, lateral release, vastus medialis obliquus (VMO) plasty, or trochlear osteotomy. Patient charts were reviewed for data collection. Follow-up was obtained in 6 weeks and 4 months postoperatively, in case of fusion without further complications. Longer follow-up was only on indication.

### Surgical technique

**Fig. 1:**
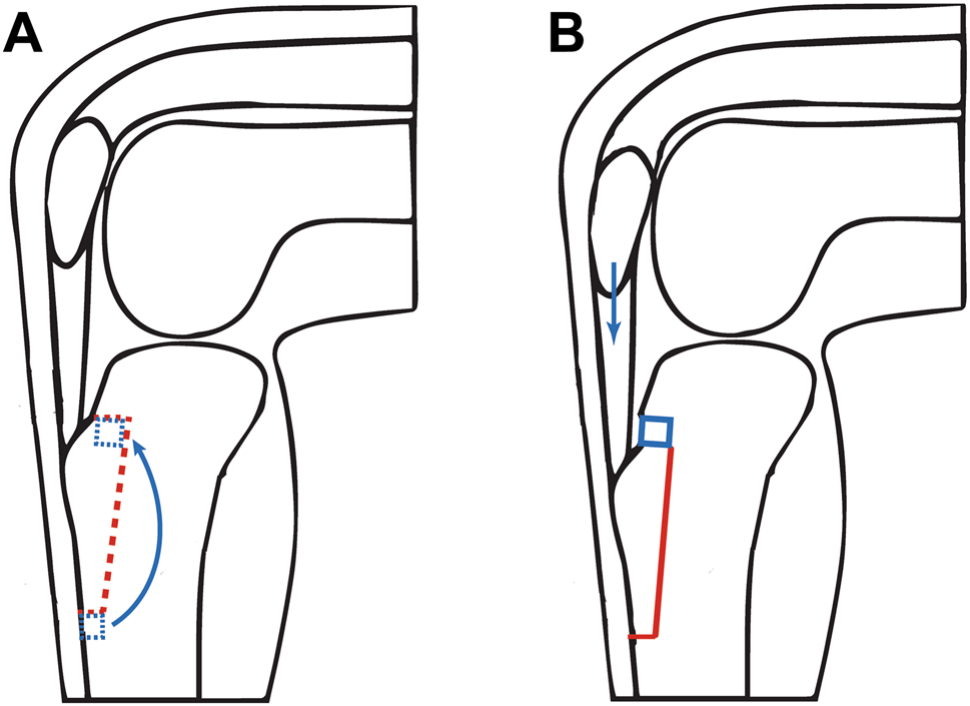
Schematic drawing of the V-shaped tibial TTO tubercle osteotomy for transfer. **a** The red dashed line reflects the cut for complete detachment of the tibial tubercle. The blue dashed line marks the small bone block that is transferred from distal to proximal. **b** Situation after distalization of the tubercle with the bone part from distal put back proximally

The tibial tubercle transfer was performed making a V-shaped osteotomy of the attachment of the patellar tendon on the tibial tubercle using a saw and osteotome (Fig. 1a), as earlier described by Caton and van de Groes [7, 12].

Through an anteromedial approach, the patellar tendon is identified and the periosteum is released. The tibial tubercle is completely detached on three sides with an oscillating saw and osteotome to perform a distal transfer.

A piece of bone from the tibia is removed to correct the Caton–Deschamps index to 1 as planned preoperatively. This bone block was placed in the gap on the proximal side to enhance stability and to provide a more stable situation of the tuberosity (Fig. 1b). The osteotomy was fixed using two small fragment lag screws. The screws were countersunk and not placed in the same line to prevent breakage of the tubercle and irritation of the screw heads. Stable compression was obtained.

### Aftercare

Postoperative care consisted of a removable long leg plaster, cast with the knee in full extension for a 6-week period, until 2014. From 2014 to present, no cast is used. Only partial weight bearing (50%) was allowed in this period. No brace was used, but all patients were instructed to bend the knee up to 70°. If there were no complications after 6 weeks, full weight bearing and full range of motion were allowed.

### Data analysis

Complications related to the surgical procedure were classified as minor or major, according to the criteria used in Payne’s review article [9]. Major complications were defined as tibial fractures, non-union, neurovascular complications, infection, and wound complications that required surgical intervention. Minor complications include events that are unlikely to have influenced the functional outcome or caused no permanent harm to the patient.

### Statistical analysis

Descriptive statistics were used to analyse the frequency of complications as a percentage of the total. A Chi-square test was performed to look at the differences in the male-to-female ratio, and an unpaired *T* test to look at the differences in the age between the groups with and without complications.

## Results

Two hundred and sixty-three (263) knees in two hundred and three (203) patients were included. Descriptive statistics are displayed in Table 1. The median age of operation was 19 years (range 12–49 years). Most patients were female (73.8%).

**Table 1:** Descriptive statistics

Patient characteristics	
Number of patients	203
Number of knees	263
Mean age (range)	20.5 (12–49)

	*N* patients (%)
Female	194 (74)
Additional procedures performed	
None	123 (46)
VMO plasty	51 (19)
Trochlear osteotomy	50 (19)
Lateral release	16 (6)
MPFL reconstruction	7 (3)
Combined	80 (30)

Median follow-up was 4 months (range 3–120 months), because standard follow-up was only up to 4 months if uncomplicated. Most frequent reasons for longer follow-up were: recurrent dislocations, postoperative complications, consultation for contralateral knee issues, and request for TTO hardware removal.

An overview of which specific additional procedures performed can be found in Table 1. Out of the 263 knees, 144 (54.8%) had at least one additional procedure to the TTO. There was no significant difference in the age between patients with and without complications (*p* = 0.80), but the amount of women in the group with complications was higher compared to the group without complications (Chi-square= 4.5765, *p* = 0.03).

### Major complications

Thirteen knees (4.9%) had a major complication. An overview of complications is displayed in Table 2. Two patients (0.76%) sustained a tibial shaft fracture at the side of the step-cut performed during the transfer surgery: the first patient while jumping on one leg during rehabilitation 2.5 months after the surgery, and the other after 6.5 months after a fall. Both fractures were stabilized with a locking compression plate. There were two cases in which there was a problem with the part of bone removed from the distal side that was pressed into the proximal part of the osteotomy. In one patient, the bone block became a loose body that was removed arthroscopically. In the second case, this bone block was malpositioned directly underneath the patellar tendon and caused tendinopathy, and was surgically removed. There was one case of septic arthritis (0.38%) and one with a non-union (0.38%). The patient with a non-union was re-operated after 9 months. A fibrous layer on the V-shaped fragment was excised and a third screw was placed to increase stability, and this resulted in consolidation after 5 months. Proximalisation of the tubercle without screw breakage was seen in three patients (1.14%), and this was recognized after 10 days, 3 weeks, and 3 months, respectively; all three patients had the screws revised after which the osteotomy fully consolidated. In one patient, the malunion was seen after 4 years after a recurrent patellar dislocation. The bone was healed, but during the growth, the screws were pulled oblique, so the tubercle proximalised again. A correction TTO was performed. Screw breakage occurred only once, which was discovered 6 months after the surgery, but with the consolidation of the osteotomy and a Caton index of 1.1, no further action was needed. In one case, the tibial tubercle fractured 3 days after surgery because of an epileptic insult with maximum quadriceps contraction, so it was fixated with a small buttress plate.

**Table 2:** Occurrence and demographics of complications in TTO

Results	*N* (%)
Major complication	13 (4.9)
Fracture	3
Tibial shaft	2
Tibial tubercle	1
Non-union or malunion	6
Delayed union	4
Non-union	1
Malunion	1
Malposition bone block	2
Septic arthritis	1
Minor complication	5 (1.9)
Thromboembolic event	2
Wound infection	1
Delayed union	1
Delayed FROM	1

### Minor complications

In five knees (1.9%), minor complications occurred (Table 2). Two patients (0.76%) had a thromboembolic event. The other three complications occurred only once (0.38%): a superficial wound infection with a *S*. *aureus* for which a patient got antibiotics for 6 weeks, a deep flexion contracture of 90° which was restored without further surgery after 5 months to 130°, and a delayed union. The last patient had to wear an extension brace with restricted flexion without resistance up to 60° until 5 months postoperatively, after which the osteotomy consolidated. No cases of persisting disability in the range of motion were seen.

### Hardware removal

Twenty-two knees had the screws removed because of pain or irritation (8.4%).

## Discussion

**Fig. 2:**
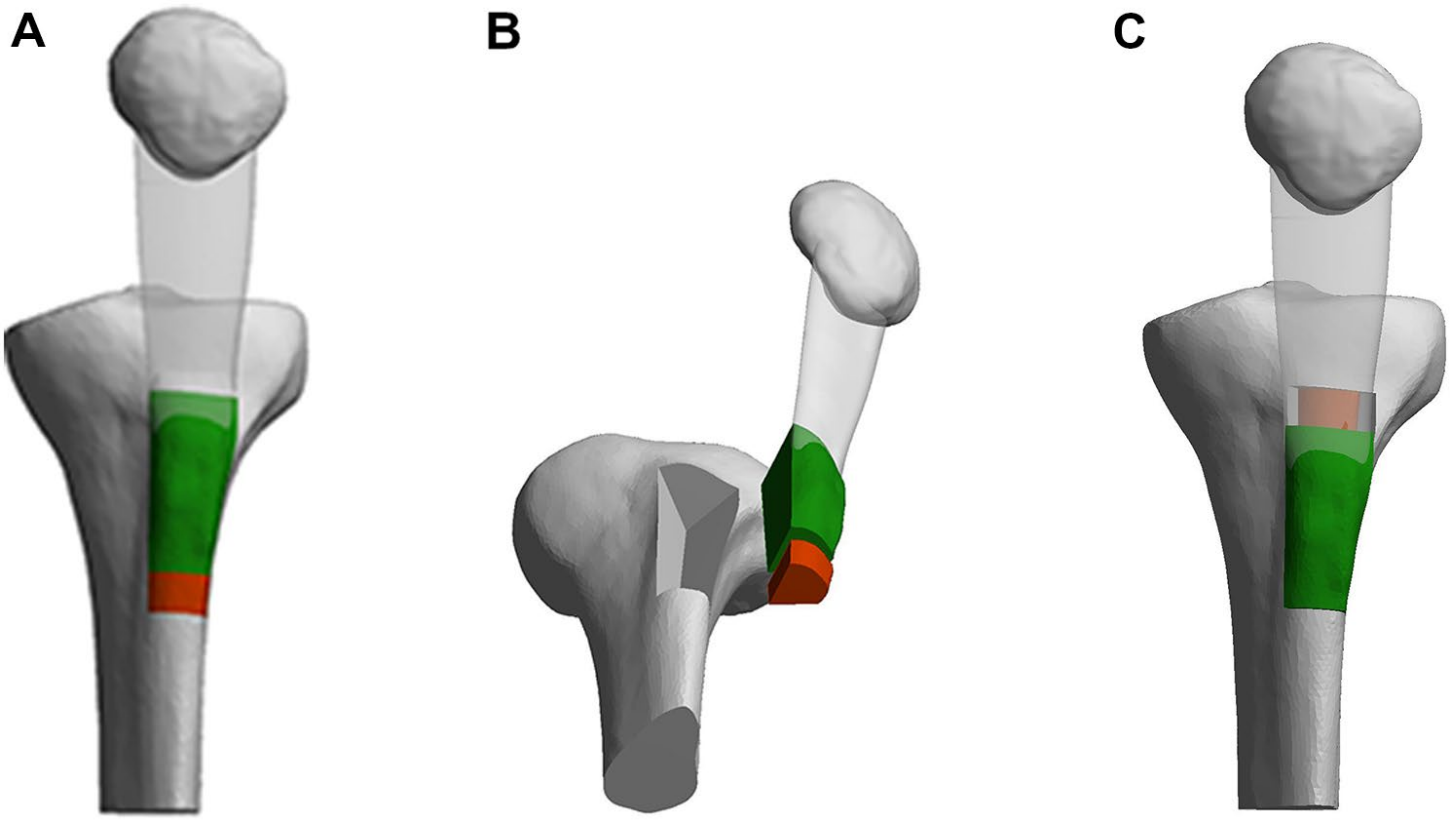
Three-dimensional schematic imaging of the TTO technique before (**a**), during (**b**), and after (**c**) the V-shaped osteotomy

The major findings of this study are the lower incidences of non-union and tibial fractures. Kanamiya et al. suggested that when a complete detachment of the tibial tubercle is performed and the medial, lateral, and distal periosteum is transected, it leads to a complete arrest of the blood flow and a higher chance of non-union [8]. From our data, we cannot confirm this theoretical concept in practice. Compared to the non-union rate of 0.8% in the 787 TTO’s published by Payne et al. [6] in their systematic review, the incidence in our group (0.38%) is even lower. This could be due to the bigger contact area of the V-shaped osteotomy with the trabecular bone for better bone healing (Fig. 2).

Secondly, the tibial fractures were only seen in 0.76%, again less than reported by Payne et al. [6] (2.4% when using a detached TTO) or Luhmann et al. [10].

There were no early tibial fractures. After the second tibial fracture, the aftercare was changed where instead of 50%, only 10% of the weight bearing was allowed for 6 weeks. QueryAlthough both the tibial fractures were seen after first 6 weeks, we think that protecting the tibia in the first stadium of bone healing will give less excessive stress on the damaged cortex at the distal cut, which is perpendicular to the shaft, and so prevents the tibial shaft fractures. In both cases, the piece of bone that was resected was not placed back proximally, because it did not fit. This might have caused a lack of stability, which could be the reason for the tibial shaft broke. Secondly, it is very important to make the distal cut carefully and not too far into the cortex of the tibia. If this happens, this will be the weak spot for stress rising.

In three cases, the tibial tubercle proximalised without breakage of the screws. It has been recommended that these screws should be at least 2 mm longer than the measured bi-cortical distance to ensure adequate bite [13]. In retrospect, this was not the case in two out of three situations.

In only one case, the piece of bone that was transpositioned from distal to proximal became loose. Therefore, no additional fixation is necessary for this bone block besides compression between the cortices.

The infection rate was 0.76% with one septic arthritis and one superficial wound infection, comparable to the findings of Payne et al. [6].

The other remarkable finding was the lower number of screw removals in this case series. Most studies maintain percentages up to 50% of the hardware removal in TTO. Payne found that in the complete tubercle detachment group, this risk was 48.3%. One of the reasons why, in this study, this percentage is as low as 8.4% is that we use the countersink when placing the screws. Moreover, all patients got instructed that the hardware is only removed in case of specific complaints of the screws.

The type of TTO used in this study is an osteotomy in three planes. This is technically more demanding than osteotomies in a single plane. However, because the osteotomy is performed in an open procedure, the cuts can be perfectly visualised which makes it less complex.

One of the reasons for the low complication rates could be that two experienced surgeons who are very familiar with the procedure did the surgery. For instance, making the distal cut very carefully is crucial. If this is too deep, chances of fracturing the tibia could theoretically rise. The complications which we had in this series were not only in the beginning of the study, but spread during the years investigated. We conclude that the learning curve is not so long. This study, however, points out that in experienced hands, this type of TTO is a relatively safe technique.

Divano et al. [14] evaluated TTO in total knee arthroplasty (TKA), both primary and revision procedures. Their results showed that the use of a TTO in total knee arthroplasty did not influence the knee scoring and function, with a union rate close to 100%. They concluded that when adequate exposure cannot be obtained, step-cut TTO is a safe and reproducible procedure if strict attention is paid to technique and fixation. It does not compromise the functional results of TKA.

The most important weakness is the retrospective nature of this study, using only available patient charts. Because of the highly specialized character of our clinical practice in patellofemoral instability, the chances that complications occurred without our knowledge are small. All patients were followed up until at least 4 months, so wound problems or non-unions would have been detected. It is very unlikely that tibial fractures were treated in another clinic. A distinct advantage of this study is its large sample size and the uniform technique that was used.

## Conclusion

A V-shaped TTO is a (relatively) safe procedure with a low complication rate. The risks on non-union and tibial fractures are particularly low despite the complete detachment of the periosteum and usage of step-cut osteotomy.
